# Influence of spin-off decision on financing risk: Empirical insight from Indonesian Islamic banks

**DOI:** 10.12688/f1000research.157435.2

**Published:** 2025-02-04

**Authors:** Zulfikar Bagus Pambuko, Jaka Sriyana, Akhsyim Affandi, Abdul Hakim

**Affiliations:** 1 Universitas Islam Indonesia, Yogyakarta, 55598, Indonesia; 2 Universitas Muhammadiyah Magelang, Magelang, 56172, Indonesia

**Keywords:** Islamic Banks, Non-Performing Financing, Financial Resilience, Government Policy

## Abstract

**Background:**

Spin-offs play a significant role in organizational development strategies, particularly in Islamic banking, by fostering entrepreneurship, innovation, and Shariah-compliant management practices. Indonesia stands as a pioneer in implementing the dual banking system and has established a spin-off policy to foster the growth of Islamic banking. This study investigates whether the spin-off decision has a significant impact on financing risk in Indonesian Islamic banks.

**Methods:**

Financing risk is measured by the non-performing financing ratio, while the spin-off decision is represented by a dummy variable equal to 1 for the post-spin-off period and 0 for the pre-spin-off period. This study utilizes data from semi-annual reports of 35 Indonesian Islamic banks and analyzes it using a dynamic panel model with the Generalized Method of Moments (GMM).

**Results:**

The findings reveal that spin-offs significantly reduce financing risk, thereby enhancing the financial resilience and boosting investor appeal. Notably, this implies that Islamic banks operating as Islamic windows exhibit a higher level of financing risk compared to fully-fledged Islamic banks. Furthermore, a noteworthy pattern emerges that spin-off Islamic banks with substantial assets demonstrate greater risk in comparison to their counterparts with more modest assets. System GMM also confirmed the result.

**Conclusions:**

Islamic banks can significantly reduce their financing risks by establishing independent Islamic banks, or spin-offs. Unlike Islamic windows, which are typically integrated within conventional banks and face higher risk levels, standalone Islamic banks offer greater flexibility and control over their operations.

## Introduction

Islamic banking has emerged as a vital component of the global financial landscape, offering a unique alternative based on principles grounded in ethical and Shariah-compliant practices ( Al Ansari & Alanzarouti, 2020; Jaafar & Brightman, 2022). Islamic banks dominate 70% of the total assets in the global Islamic finance sector, signifying a growth of 17% compared to the previous period. The primary driving force behind this accomplishment is the significant contribution of countries with Muslim-majority populations, particularly in the GCC, MENA, and Southeast Asia regions. This progress is primarily supported by effective governance and well-suited policies ( ICD IsDB, 2022). In Indonesia, a prominent Muslim-majority nation, the Islamic banking sphere has showcased notable expansion and adaptability, establishing its role as a crucial element within the nation’s financial framework ( Trinugroho et al., 2018). Within this period of growth, the spin-off policy has been contemplated as a strategy to navigate the complexities of contemporary banking while adhering Shariah principles ( Pambuko & Sriyana, 2023; Trinugroho et al., 2021).

The phenomenon of spin-off becomes the one of the key dynamics influencing the financial performance of Indonesian Islamic banks ( Pambuko & Sriyana, 2023; Trinugroho et al., 2021). As a strategy, this refers to the process of establishing separate entities from the parent institution, often with distinct business focuses ( Chai et al., 2018). According to Article 68 of Law 21 of 2008, all Islamic windows are required to undergo a spin-off after 15 years or if their assets have exceeded 50% of their conventional parent bank’s assets ( Nurkarim et al., 2021; Pambuko, 2019; Rokhmawati et al., 2022). These spin-offs were achieved through various methods, including pure spin-offs, conversions, mergers, and acquisitions ( Al Arif, Masruroh, et al., 2020a). The rationale behind such spin-offs often involves streamlining operations, improving risk management, optimizing organizational structures, enhancing efficiency, fostering growth, and complying with sharia law ( Al Arif, 2014; Al Arif et al., 2018; Hamid, 2015).

The Financial Services Authority of Indonesia (2023) reports a significant increase in Islamic banking institutions as of May 2023. There are now 13 Islamic commercial banks and 20 Islamic windows, compared to just 5 commercial banks and 27 windows before the spin-off policy’s implementation. This law, enacted in 2008, mandated all Islamic windows to become full-fledged Islamic banks by 2023 ( Pambuko et al., 2024). However, many Islamic windows haven’t completed this process, highlighting the need for further study on how spin-offs impact Islamic banking performance and their adherence to Sharia principles. Furthermore, the issue of spin-offs has also become one of the key strategies to enhance the contribution of Islamic banking in other countries. As reported, Pakistan, Bahrain, Kuwait, and Afghanistan are among the countries earnestly implementing this issue in the development of Islamic banks ( ICD IsDB, 2022). Therefore, Indonesia, as a pioneer of the implementation of spin-off strategy in the Islamic banking sector, deserves to be a benchmark in the implementation of this strategy, particularly as an effort to sustain the Islamic banking industry.

In the last decade, many researchers have directed their attention toward identifying the impact of spin-off policies on the performance of Islamic banks. Several indicators of financial performance have been focused on, including profitability ( Hamid, 2015; Nurkarim et al., 2021), efficiency ( Al Arif et al., 2018; Al Arif, Mufraini, et al., 2020b; Al Arif & Nabilah, 2022; Hadziq et al., 2022; Pambuko, 2019; Rusydiana et al., 2019), market share ( Al Arif, 2017; Al Arif et al., 2023), asset growth ( Al Arif, 2015a; Al Arif et al., 2017), financing growth ( Al Arif, 2015b), deposit growth ( Al Arif, 2014, 2018b, 2018a; Al Arif et al., 2024), and market power ( Rayyani et al., 2022). While there’s been a lot of research on spin-offs, how they affect a bank’s risk of financing risk (bad loans) has not been well-studied. This is an important gap to address, especially considering the recent Basel III framework from the Basel Committee on Banking Supervision (BCBS). These new global regulations aim to make banks worldwide more stable by considering all their risks, both from loans they’ve made (on-balance sheet) and from their commitments (off-balance sheet exposures) ( Abdurraheem et al., 2023).

This scientific paper aims to bridge these knowledge gaps by offering an empirical analysis of the relationship between spin-off decision and the prevalence of financing risk in the Indonesian Islamic banking sector. While previous research suggests spin-off decisions can improve financial performance, the potential effect on financing risk has been overlooked. This study will address this gap by examining how spin-offs influence financing risk. By untangling these linked factors, we aim to provide valuable insights for both academics and Islamic bankers, fostering a deeper understanding of how strategic restructuring impacts the financial soundness of Islamic banks. Moreover, given the intricate relationships between spin-off strategies and financing risk, policy implications and strategic recommendations stemming from this study could serve as a catalyst for informed decision-making within the Islamic banking industry.

## Literature review

The management of bad financing or non-performing financing (NPF) presents a critical challenge for Islamic banking institutions in Indonesia ( Imaduddin, 2008; Nugroho et al., 2018; Soekapdjo et al., 2018). Non-performing financing, characterized by loans or financing contracts in default or at risk of default, can have far-reaching implications on the stability and credibility of financial institutions ( Hernawati et al., 2021). In traditional banking, its known as non-performing loan (NPL). The existence of financing risk can hinder the stability of Islamic banking entities, potentially jeopardizing their capacity to facilitate economic growth and foster financial inclusivity ( Banna et al., 2022). Considering the distinct characteristics of Islamic finance, rooted in risk-sharing and asset-backed principles, a comprehensive exploration of how spin-off decisions influence financing risk, specifically non-performing financing, requires further investigation.

### Bank-specific factors influencing financing risk

Research on NPF in Islamic banking and NPL in conventional banking reveals that various internal and external factors influence financing risk. For conventional banks, external factors such as macroeconomic conditions play a dominant role in shaping NPL levels, while in Islamic banks, both internal and external factors significantly affect NPF ( Loang et al., 2023; Setiawan et al., 2017). Among bank-specific factors, an increase in NPF is associated with variables such as bank size, operating efficiency ratio, liquid asset ratio, financing-to-deposit ratio, and foreign ownership ( Havidz & Setiawan, 2015; Hosen & Muhari, 2019; Osunkoya et al., 2023; Rahim & Zakaria, 2013; Saw et al., 2022; Zheng et al., 2019). Conversely, factors that contribute to a reduction in NPF include higher net operating profit, capital adequacy ratio, return on assets, cost-to-income ratio, deposit fund growth, return on equity, loan growth, and market concentration ( Hartanto & Samputra, 2023; Hosen & Muhari, 2019; Muhammad et al., 2020; Rahim & Zakaria, 2013; Zheng et al., 2019).

### Macroeconomic factors influencing financing risk

Macroeconomic conditions also significantly impact the level of financing risk in Islamic banks. Negative economic indicators such as exchange rate fluctuations, inflation, and economic policy uncertainty have been found to increase NPF levels ( Firmansyah, 2014; Hosen & Muhari, 2019; Karadima & Louri, 2021; Osunkoya et al., 2023; Zheng et al., 2019). On the other hand, positive macroeconomic trends such as GDP growth and reduced unemployment contribute to a decrease in NPF ( Ferhi, 2018; Hassan et al., 2019; Hernawati et al., 2021; Le & Diep, 2020; Zheng et al., 2019). Additionally, a surplus in foreign exchange reserves can trigger a financing boom, which may subsequently affect financing quality and contribute to increased NPF levels ( Kuncoro, 2024).

## Methods

To examine the impact of spin-off decisions on financing risk, this study utilizes semi-annual bank-level data from 2006 to 2022 for Islamic banks in Indonesia. The bank-specific data, derived from the financial statements of 35 Islamic banks, were acquired from the Islamic Banking Statistics which is reported by Indonesian Financial Services Authority (FSA). The sample consists of 16 full-fledged Islamic banks and 19 Islamic windows. Macroeconomic data were sourced from Statistics Indonesia, Bank Indonesia, and OPEC basket price. We employed an unbalanced panel dataset encompassing 1032 observations.

The Indonesian Islamic banking landscape has witnessed various restructuring strategies. Pure spin-offs were employed by banks such as Bukopin Syariah and BNI Syariah in 2008, BRI Syariah in 2010, BJB Syariah in 2010, and Bank Nano Syariah in 2024. Conversion, another common approach, was adopted by BTPN Syariah in 2014, Bank Aceh Syariah in 2016, BPD NTB Syariah in 2018, and BPD Riau Kepri Syariah in 2022. The formation of Bank Syariah Indonesia in 2021 marked a significant merger in the sector. Moreover, a combination of acquisition and conversion was utilized sequentially by banks including Bank Panin Dubai Syariah in 2009, Bank Victoria Syariah, BCA Syariah, and Bank Aladin Syariah in 2010.

Table 1 explains the data we used in this study. We looked at the ratio of non-performing financing (NPF) to total financing, which is a common measure of bad loans in Islamic banks ( Saw et al., 2022). Spin-off is a proxy for spin-off decisions, represented by a dummy variable where 1 denotes post-spin-off time and 0 signifies pre-spin-off time. The bank-specific factors include bank size (log total assets of each Islamic bank) and market share, assessed by the individual Islamic bank’s asset over the total asset industry of Islamic banks. To understand the economic climate, we looked at the Consumer Price Index (CPI) for inflation and the Industrial Production Index (IPI) for economic growth. The variable OIL is based on the OPEC basket price, which represents the per-barrel oil price. This price serves as a crucial benchmark in determining crude oil prices in the global market, including Indonesia, although it is merely one of many indicators influenced by various complex factors.

**Table 1.** Regression variables.

Variable	Description	Source
NPF	Non-performing financing is the ratio of bad financing by total financing	Islamic banking statistics
Spinoff	Dummy variable, it is one if the period after spin-off decision, zero otherwise	Banks’ profile
Bank size (SIZE)	Natural logarithm of total assets	Islamic banking statistics
Market share (MS)	The ratio of Islamic banks' assets to the industry's overall assets	Islamic banking statistics
CPI	Inflation is measured using the Consumer Price Index (CPI), which compares the total cost of a fixed basket of goods and services in the current period to the cost of the same basket in a base period	Bank of Indonesia
IPI	Economic growth is measured using the Industrial Production Index (IPI), which is calculated by dividing the value of production in a specific period by the value of production in the base year 2010	Statistics Indonesia
Oil Price (OIL)	A weighted average price of crude oil blends produced by the OPEC member countries	OPEC basket price

This study utilizes a dynamic panel regression model with individual fixed effects to assess the impact of spin-off decisions on financing risk for Islamic banks in Indonesia. The dynamic panel approach addresses both unobserved bank-specific characteristics and potential endogeneity issues that might bias the results. To further account for potential simultaneity and correlation between variables, the study employs the Generalized Methods of Moment (GMM) estimation technique. Equation (1) details the empirical model used in this analysis, which incorporates the spin-off decision as the main independent variable. Additionally, control variables such as bank size, market share, and macroeconomic factors like inflation, economic growth, and oil price are included to account for their potential influence on financing risk.

$$Y_{\mathrm{it}}=\alpha +\beta_{1}\;Y_{\mathrm{it}-1}+\beta_{2}\;{\text{Spinoff}}_{t}+\beta_{3}\;{\text{BankSpecific}}_{i,t}+\beta_{4}\;{\text{MacroEconomic}}_{t}+\varepsilon_{i,t}$$ (1)

Where *Y _it_* is the non performing financing (NPF) for bank *i* and year *t*; *Spinoff _t_* is a dummy variable, it is one if the period after spin-off decision, zero otherwise; *BankSpecific _i,t_* is control variable consisting of bank size dan market share; *MacroEconomic _t_* is control variable consisting of inflation, economic growth, and oil price; and *ε _i,t_* is the error term. The first lag of the dependent variable has been integrated into the model as an independent variable to address the possible persistence of NPF changes in time. Our analyses utilized a difference GMM Arellano-Bond estimator. To ensure robustness, we conducted a two-step System GMM estimator for further validation.

Then we analyze the impact of bank size on the interaction between spin-off decisions and non-performing financing. We created an interaction variable in the form of Spinoff _t_*Size _it_ for this purpose. The equation (2) represents the estimation model.

$$Y_{\mathrm{it}}=\alpha +\beta_{1}\;Y_{\mathrm{it}-1}+\beta_{2}\;{\text{Spinoff}}_{t}+\beta_{3}\;{{\text{Spinoff}}_{t}}^{*}{\text{Size}}_{\mathrm{it}}+\beta_{4}\;{\text{BankSpecific}}_{i,t}+\beta_{5}\;{\text{MacroEconomic}}_{t}+\varepsilon_{i,t}$$ (2)

Data analysis was conducted using EViews 12 by S&P Global. Prior to interpretation, diagnostic tests were performed. The Sargan test was employed to assess the validity of the instruments, while the Arellano-Bond test was used to detect the presence of autocorrelation. A lag order of one (lag 1) was used for estimation. A well-specified model should exhibit valid instruments and no autocorrelation.

## Results and Discussion

### Descriptive analysis

Table 2 presents descriptive statistics for the variables spanning from 2006 to 2022. The average value of NPF, as a financing risk proxy, stands at 3.10%, remaining below the generally accepted upper limit of 5.00%. The lowest NPF value, at 0.00%, indicates that some Islamic banks exhibit no risky financing, while the highest recorded NPF value is 53.27%. This relatively high variability is supported by a standard deviation of 4.17%. SPINOFF, a dummy variable, has a mean of 0.27, indicating that spin-off events are relatively rare. Its standard deviation of 0.45 suggests moderate variability in occurrence. Regarding assets and market share, the lower mean values compared to the standard deviation suggest that Islamic banks in Indonesia are still dominated by a few larger banks, reflecting differences in institutional capacity and market reach.

**Table 2.** Descriptive analysis of variables.

	Obs.	Mean	Max	Min	Std. Dev.
SPINOFF	1032	0.27	1.00	0.00	0.45
NPF	1032	3.10	53.27	0.00	4.17
SIZE (IDR million)	1032	9579281.35	305727438.00	3176.00	22725240.62
MS	1032	3.23	40.13	0.01	6.39
CPI	1032	4.90	15.53	1.33	2.70
IPI	1032	122.54	151.76	95.71	18.33
OIL (IDR million/barrel)	1032	0.86	1.70	0.34	0.28

Similarly, the Consumer Price Index (CPI) and Industrial Production Index (IPI) show moderate variability, with standard deviations of 2.70 and 18.33, respectively, reflecting macroeconomic conditions that influence Islamic banking performance. Oil prices (OIL), a critical variable for resource-dependent economies, have a mean of IDR 0.86 million per barrel, with relatively stable fluctuations (standard deviation of 0.28). This stability contrasts with the high variability observed in institutional size and non-performing financing.

Overall, the descriptive analysis underscores significant heterogeneity in institutional characteristics and economic factors, suggesting the need for tailored strategies in managing Islamic banks. Institutions must address wide disparities in size and market share, ensuring resilience to external economic conditions such as inflation, industrial output fluctuations, and oil price volatility.

### Empirical results

The analysis commenced by identifying the issue of multicollinearity among the independent variables. Table 3 displays the correlation scores among the independent variables. A correlation score of < 0.8 was indicative of the absence of multicollinearity ( Mukhibad, Nurkhin et al., 2023b; Rizvi et al., 2020). The highest correlation between independent variables was 0.5678 (correlation between SIZE and IPI), followed by SIZE and MS (0.5592). These findings signify the absence of multicollinearity within the model.

**Table 3.** Correlation matrix.

	SIZE	MS	CPI	IPI	OIL
SIZE	1				
MS	0.5592	1			
CPI	-0.3750	0.0442	1		
IPI	0.5678	-0.0440	-0.5417	1	
OIL	0.1693	-0.0254	-0.0330	0.2726	1

The subsequent analysis examines the impact of spin-off decisions on the financing risk of Islamic banks. Table 4 presents the outcomes of the difference GMM and system GMM. Difference GMM estimator is depicted in regressions (1)-(4), while system GMM estimator in regressions (5)-(8). Regressions (1) and (5) only incorporate bank-specific variables, consisting of assets (SIZE) and market share (MS). In regressions (2) and (6), we include additional control variables such as inflation (CPI), economic growth (IPI), and global oil prices (OIL). In regressions (3), (4), (7), and (8), we introduce interaction variables, specifically spin-off*size. Towards the end of the table, it is indicated that no issues of instrumental validity and autocorrelation were found. To elaborate, the results of the Sargan test reject the null hypothesis, confirming the validity of the instruments. Furthermore, the results of the Arellano-Bond autocorrelation test, AR(2), also reveal the absence of autocorrelation in the model.

**Table 4.** Determinants of Islamic banks’ performance.

	Difference GMM				System GMM			
	(1)	(2)	(3)	(4)	(5)	(6)	(7)	(8)
NPF(-1)	0.3599 * (1385.50)	0.3468 * (349.62)	0.3555 * (593.07)	0.3408 * (134.95)	0.3768 * (2221.58)	0.3737 * (119.03)	0.3761 * (172.18)	0.3687 * (90.03)
spinoff	1.1110 * (380.49)	0.6900 * (63.40)	-31.4797 * (-172.45)	-31.3797 * (-40.51)	-1.5722 * (-1614.53)	-1.6502 * (-50.65)	-19.8628 * (-95.16)	-23.4687 * (-39.38)
spinoff *size			1.1062 * (165.72)	1.0860 * (37.81)			0.6136 * (75.08)	0.7271 * (36.60)
SIZE	0.1351 * (58.24)	0.2951 * (17.06)	-0.1487 * (-34.82)	0.0808 ** (2.49)	0.2491 * (1814.80)	0.2059 * (6.34)	0.1836 * (35.65)	0.2364 * (7.82)
MS	0.1760 * (239.99)	0.1574 * (20.22)	0.1327 * (32.35)	0.1042 * (8.22)	0.0349 * (57.10)	0.0366 ** (2.37)	0.0286 * (7.65)	0.0327 *** (1.66)
CPI		0.0038 * (4.40)		0.0002 (0.10)		0.0035 * (4.58)		0.0061 * (3.01)
IPI		-0.0002 (-0.10)		-0.0065 *** (-1.75)		0.0106 * (4.66)		0.0047 *** (1.72)
OIL		-1.1527 * (-97.11)		-1.2491 * (-49.54)		-1.2242 * (-36.87)		-1.4065 * (-34.90)
Sargan test	0.3890	0.3166	0.4097	0.3601	0.3991	0.3155	0.3998	0.3029
AR(1)	0.0000	0.0251	0.0297	0.0256				
AR(2)	0.1660	0.1532	0.1452	0.1467				
No. of Obs.	948	948	948	948	948	948	948	948

Notes: NPF(-1) = lagged non-performing financing; spinoff= dummy variable as a spin-off time decision; spinoff*size = interaction variable of spin-off time decision and bank size; Size=natural logarithm of total assets; MS= asset of individual Islamic bank compared by total asset of Islamic banking industry; CPI = inflation; IPI = economic growth; OIL= natural logarithm of OPEC oil price. p-values in parentheses

* p<0.01 .

** p<0.05 .

*** p<0.1.

The findings from the analysis provide compelling evidence regarding the influence of the lagged dependent variable, NPF(-1), which consistently exhibits a statistically significant positive effect across all models at the 1% significance level. This outcome substantiates the notion that the financing risk experienced in the preceding period contributes to an increase in the non-performing financing ratio during the present period. This result underscores a significant challenge in the Islamic banking sector, suggesting that the mechanisms in place to address the issue of bad financing have not yet achieved optimal effectiveness. This aligns with previous research conducted by Mukhibad, Jayanto et al. (2023a), who reached similar conclusions in their study. Notably, the finding underscores the persisting complexity of managing NPF in Islamic banking and emphasize the need for more refined strategies to mitigate such risks. Interestingly, the result contrasts with those of Hartanto & Samputra (2023), whose research indicated a potential improvement in the non-performing financing situation from period t-1 to period t. This variation emphasizes the intricate and many-sided aspects of how financing risk behaves in Islamic banking.

Regarding the main focus of this study, it becomes apparent that the spin-off decisions undertaken by Islamic banks wield a substantial and statistically significant impact, consistently evident at the 1% significance level across all models. This pervasive influence, however, diverges in models (1) and (2). Specifically, the majority of models reveal a noteworthy negative effect resulting from the strategic choice of Islamic banks to embark on spin-offs. This intriguing discovery underscores a pivotal insight – that the judicious adoption of spin-off strategies by Islamic banks yields a salutary outcome, effectively tempering the levels of financing risk. This outcome resonates with the emerging understanding that Islamic banks’ autonomy in resource management holds the potential to substantially shape their risk profile. This profound effect echoes the findings of a distinct study, where a compelling revelation was made that full-fledged Islamic banks exhibit a more adept ability to mitigate non-performing financing as compared to Islamic windows ( Pambuko & Pramesti, 2020; Pernamasari, 2020). These findings collectively affirm the advantageous influence of spin-off decisions in fostering improved risk management practices and enhancing the overall stability of Islamic banking entities.

We also investigated the impact of size on financing risk. The analysis results reveal that size has a significant positive impact on non-performing financing, except in model (3) where it has a negative impact. The finding indicates that an increase in the asset size will increase financing risk. The result supports the finding of Hosen and Muhari (2019) and Saw et al. (2022). It is also validated the ‘bad management theory’ proposed by Berger and DeYoung (1997). Within this theoretical framework, the orchestration of poor management practices within banking institutions leads to lower loan quality and increases the level of non-performing loans. Furthermore, these results are corroborated by the estimations of the interaction variable (spinoff*size), which significantly influences non-performing financing. Thus, larger Islamic banks that undergo spin-offs carry a higher financing risk compared to smaller banks. As highlighted by Trinugroho et al. (2021), the multifaceted nature of financing contracts in Islamic banking substantially elevates the risk of non-performing financing.

Further analysis concerning the impact of market share on the financing risk of Islamic banks was conducted. Across all models, a positive relationship between market share and non performing financing is revealed, except in models (6) and (8), where the relationship is significant at the 5% and 10% levels, respectively. The finding strengthens the previous estimations indicating that greater asset dominance increases the potential for non-performing financing. As for the control variables, we only found robust and negatively correlated estimates for NPF and OIL. This negative relationship explains that an increase in global oil prices will decrease financing risk for Islamic banks. With a certain level of oil price increase, related product prices become more competitive, stimulating business sectors and lowering financing risk. As Abimanyu et al. (2023) pointed out, Indonesia’s policy aims to ensure that global oil price movements contribute positively to the state’s finances and trade, while minimizing inflationary risks. The result also supports the findings of Ryandono et al. (2022) dan Al Jabri et al. (2022).

On the other hand, economic growth and inflation exhibit inconsistent results across different model specifications. Inflation generally exerts a positive influence on financing risk, as observed in models 2, 6, and 8. This finding aligns with previous studies, which suggests that inflation can reduce purchasing power, leading to increased loan defaults ( Awdeh et al., 2024; Patiu & Eleazar, 2024). However, the impact of economic growth on financing risk is more nuanced, with negative effects observed in the difference GMM estimation and positive effects in the system GMM estimation. This discrepancy might be attributed to the non-linear relationship between economic growth and NPF, where difference GMM captures short-term effects while system GMM captures long-term effects. The positive relationship in the system GMM, which is considered to provide more robust estimates, may reflect the potential for credit booms during periods of economic expansion, leading Islamic banks to loosen lending standards ( Castro, 2013; Huizinga & Laeven, 2019; Mongid et al., 2023). Furthermore, this divergence in results may be explained by the finite sample bias in difference GMM, which relies solely on differences across time periods and excludes the level data.

## Conclusion

This study investigates the impact of spin-off decision – the separation of Islamic windows from their conventional parent banks to become full-fledged Islamic banks – on financing risk in Indonesian Islamic banks. We estimate over a substantial timeframe in the journey of Islamic banks in Indonesia, spanning from 2006 to 2022. Our findings reveal that Islamic windows carry a higher risk compared to full-fledged Islamic banks that undergo spin-offs. We also discover that spin-off Islamic banks with larger assets are more exposed to risk than those with smaller assets. This notice is supported by the regression that financing risk increases with the growth of assets and market share. In essence, our study not only contributes to the empirical understanding of Islamic banking dynamics but also advances the discourse surrounding prudent risk management strategies in financial systems governed by ethical and Shariah-compliant principles.

These findings yield several policy implications. We have strong evidence that the independence of Islamic banks makes them more sensitive to risk and adept at mitigating it. Therefore, the mandatory spin-off policy to be implemented for all Islamic windows by the end of 2023 is a viable approach. Moreover, the potential rise in market dominance by Islamic banks, which could amplify financing risk, calls for complementary policies to mitigate the potential negative outcomes.

The present study has identified several limitations. The utilization of a dummy variable to assess the impact of spin-offs on financing risk, while effective in capturing the immediate effects of regulatory policies, may not fully account for the nuanced dynamics at play. Moreover, the study’s scope is constrained by the use of semi-annual data up to 2022 and its focus on the Indonesian Islamic banking industry. To address these limitations, future research could benefit from employing a difference-in-differences analysis to more precisely estimate the causal impact of spin-offs. Furthermore, incorporating a broader range of internal and external variables, as well as expanding the analysis to include Islamic banks in Southeast Asia or OIC countries, would provide a more comprehensive understanding of the phenomenon.

### Ethical considerations

This study does not involve human participants, animal subjects, or plant specimens.

## Data Availability

Zenodo: Influence of spin-off decision on financing risk: Empirical insight from Indonesian Islamic banks https://doi.org/10.5281/zenodo.13879461 ( Pambuko, 2024). The project contains the following underlying data:
− dataset financing risk.xlsx Data are available under the terms of the Creative Commons Attribution 4.0 International license (CC-BY 4.0). The data used in this research is open-access and can be downloaded from the following website: Indonesian Banking Statistics on Financial Service Authority Website: https://www.ojk.go.id/en/kanal/perbankan/data-dan-statistik/statistik-perbankan-indonesia/Default.aspx; BPS-Statistics Indonesia: https://www.bps.go.id/en/statistics-table/2/MjA3NyMy/indeks-produksi-bulanan-industri-besar-dan-sedang-menurut-kbli-2-digit--kbli-2020---2010-100-.html; Bank Indonesia: https://www.bi.go.id/en/statistik/indikator/data-inflasi.aspx; and OPEC Basket Price: https://www.opec.org/opec_web/en/data_graphs/40.htm.

## Software availability statement

EViews 12, developed by S&P Global and available at https://www.eviews.com/home.html, is a powerful econometric software accessible to researchers based on their license type. For those seeking a free version, a student version is available for download at https://www.eviews.com/EViews12/EViews12Univ/evuniv12.html#stud.
