# Supplementary material for: Influence of spin-off decision on financing risk: Empirical insight from Indonesian Islamic banks
Source: F1000Res. 2025 Apr 28;13:1251. Originally published 2024 Oct 18. [Version 3] doi: 10.12688/f1000research.157435.3 (PMC11993850; doi:10.12688/f1000research.157435.3)
Supplement: Peer review history

# Reviewer report: Reviewer response for version 3

**DOI:** 10.5256/f1000research.180998.r381249

**Published:** 2025-05-13

**Authors:** Agus Widarjono

**Affiliations:** 1 Universitas Islam Indonesia, Yogyakarta, Special Region of Yogyakarta, Indonesia

The comments

**INTRODUCTION**

 Thank you for responding to comments and providing feedback. 

**LITERATURE REVIEW**

 Thank you for responding to comments and providing feedback. 

**METHOD AND RESULT AND DISCUSSION**

 The author failed to respond to two robustness tests:

 1. Changing variable of interest.

 There is no single measurement to measure Islamic bank financing risk as a dependent variable. Two methods are widely used, namely NPF (non-performing financing) and FLP (financing loss provision). To get robust results, the author needs to change the variable of interest. FLP is an alternative measure of financing risk. For example, see the following reference:

 Widarjono, A., Alam, M. M., Rafik, A., Afandi, A., & Sidiq, S. (2025). Nexus between competition, concentration and bank risk-taking in Indonesian Islamic banking. *International Journal of Islamic and Middle Eastern Finance and Management*, *18*(3), 672–690. (refer to 1)

 2. Changing estimation method

 The dynamic panel model faces an endogeneity problem. The dynamic panel model is estimated by the GMM method. There are two GMM estimators, namely the first difference GMM (Arellano and Bond, 1991) and the system GMM (Arellano and Bover, 1995; Blundell and Bond, 1998) to address the endogeneity issue in the dynamic panel model using instrumental variables. The system GMM extends the first difference GMM to resolve the problems of instrument weakness so the system GMM generates more robust estimators than the first difference GMM. Therefore, authors must estimate using static panel regression as an alternative method for the robustness test. Furthermore, the dynamic panel method will generate a biased and inconsistent estimator if the cross-sectional object is small. The number of objects in this study is small, consisting of 35 Islamic banks. (Al-Muharrami & Murthy, 2016), see the following references:

 Al-muharrami, S., & Murthy, Y. S. R. (2016). Interest banking spreads in Oman and Arab GCC. *International Journal of Emerging Markets*, *12*(3), 532–549. (refer to 2 )

**CONCLUSION**

 Thank you for responding to comments and providing feedback.

Is the work clearly and accurately presented and does it cite the current literature?

Yes

If applicable, is the statistical analysis and its interpretation appropriate?

Partly

Are all the source data underlying the results available to ensure full reproducibility?

Yes

Is the study design appropriate and is the work technically sound?

Yes

Are the conclusions drawn adequately supported by the results?

Yes

Are sufficient details of methods and analysis provided to allow replication by others?

Yes

Reviewer Expertise:

Islamic finance and banking

I confirm that I have read this submission and believe that I have an appropriate level of expertise to confirm that it is of an acceptable scientific standard.

## References

[1] : Nexus between competition, concentration and bank risk-taking in Indonesian Islamic banking. *International Journal of Islamic and Middle Eastern Finance and Management* .2025;18(3) : 10.1108/IMEFM-02-2024-0099 672-690 10.1108/IMEFM-02-2024-0099

[2] : Interest banking spreads in Oman and Arab GCC. *International Journal of Emerging Markets* .2017;12(3) : 10.1108/IJoEM-02-2016-0033 532-549 10.1108/IJoEM-02-2016-0033

# Reviewer report: Reviewer response for version 3

**DOI:** 10.5256/f1000research.180998.r381251

**Published:** 2025-05-12

**Authors:** Azharsyah Ibrahim

**Affiliations:** 1 Universitas Islam Negeri Ar-Raniry, Banda Aceh, Indonesia

The author has addressed all comments satisfactorily.

Is the work clearly and accurately presented and does it cite the current literature?

Partly

If applicable, is the statistical analysis and its interpretation appropriate?

Partly

Are all the source data underlying the results available to ensure full reproducibility?

Yes

Is the study design appropriate and is the work technically sound?

Partly

Are the conclusions drawn adequately supported by the results?

Partly

Are sufficient details of methods and analysis provided to allow replication by others?

Partly

Reviewer Expertise:

Islamic business, economics, banking, and finance

I confirm that I have read this submission and believe that I have an appropriate level of expertise to confirm that it is of an acceptable scientific standard.

# Reviewer report: Reviewer response for version 2

**DOI:** 10.5256/f1000research.176913.r372133

**Published:** 2025-04-14

**Authors:** Agus Widarjono

**Affiliations:** 1 Universitas Islam Indonesia, Yogyakarta, Special Region of Yogyakarta, Indonesia

**INTRODUCTION**

 This study investigates the effect of the spin-off on NPF. The spin-off policy is a policy to improve the performance of Islamic banks in Indonesia. However, the author has not explained why NPF is used as a measure of the success of the spin-off policy. It needs to be explained why NPF is a serious problem in Islamic banking in Indonesia so that this spin-off policy can reduce financing risk.

**LITERATURE REVIEW**

 Islamic bank financing is different from conventional bank financing. Islamic financing is broadly categorized as profit-loss sharing (PLS) financing and non-profit-sharing financing. PLS financings are subject to asymmetric information and moral hazard. As a result, PLS financing likely increases NPF. In this section, authors conduct the literature review regarding NPF but authors fail to address the impact of types of financing in Islamic banks on NPF. For example, see the following references :

1. Warninda T., et al., (2019) [Ref-1]
2. Sutrisno, S. et al., (2023) [Ref-2].

**METHOD**

 The difference and system GMM are a family of dynamic panel regression. The author must perform the robustness test with another estimation method. The author can employ static panel regression considering the number of banks is small (35 banks) while GMM requires a large number of banks.

**RESULT AND DISCUSSION**

 Authors need to perform some robustness tests:

 1. Changing variable of interest

 There is no single measure to calculate Islamic bank risk. To get robust results, the author needs to change the variable of interest. Financing loss provision (FLP) is an alternative measure of financing risk. For example, see the following reference:

 Widarjono A., et al., (2025) [Ref-3]

 2. Changing estimation method

 Considering that the number of small banks is 35, the author needs to estimate the model with static panel regression.

**CONCLUSION**

 In the conclusions, it is said that the spin-off policy is mandatory for every Islamic bank window. However, there is an important note from previous research that full spin-off likely decreases the performance of Islamic banks after several years of spin-off. See following references:

1. Al Arif M., et al., (2020) [Ref-4]
2. Trinugroho I, et al., 2021 [Ref-5]

Is the work clearly and accurately presented and does it cite the current literature?

Yes

If applicable, is the statistical analysis and its interpretation appropriate?

Partly

Are all the source data underlying the results available to ensure full reproducibility?

Yes

Is the study design appropriate and is the work technically sound?

Yes

Are the conclusions drawn adequately supported by the results?

Yes

Are sufficient details of methods and analysis provided to allow replication by others?

Yes

Reviewer Expertise:

Islamic finance and banking

I confirm that I have read this submission and believe that I have an appropriate level of expertise to confirm that it is of an acceptable scientific standard, however I have significant reservations, as outlined above.

## References

[1] : Do Mudarabah and Musharakah financing impact Islamic Bank credit risk differently?. *Research in International Business and Finance* .2019;49: 10.1016/j.ribaf.2019.03.002 166-175 10.1016/j.ribaf.2019.03.002

[2] : Does Financing Diversification Improve Bank Risk? Evidence From Indonesian Islamic Rural Banks. *International Journal of Economics and Finance Studies* .2023; 10.34109/ijefs.202315406 10.34109/ijefs.202315406

[3] : Nexus between competition, concentration and bank risk-taking in Indonesian Islamic banking. *International Journal of Islamic and Middle Eastern Finance and Management* .2025; 10.1108/IMEFM-02-2024-0099 10.1108/IMEFM-02-2024-0099

[4] : Market Structure, Spin-Off, and Efficiency: Evidence from Indonesian Islamic Banking Industry. *Emerging Markets Finance and Trade* .2020;56(2) : 10.1080/1540496X.2018.1553162 329-337 10.1080/1540496X.2018.1553162

[5] : Is spin-off policy an effective way to improve performance of Islamic banks? Evidence from Indonesia. *Research in International Business and Finance* .2021;56: 10.1016/j.ribaf.2020.101352 10.1016/j.ribaf.2020.101352

# Response

**Published:** 2025-04-17

**Authors:** Zulfikar Bagus Pambuko

**Affiliations:** Islamic Economics Law, Universitas Muhammadiyah Magelang, Magelang, Central Java, Indonesia

Dear Reviewer

 We would like to warmly thank you for reading our manuscript and your valuable comments which would substantially improve our paper. We have followed carefully all your comments in the preparation of the revised version of the paper. Our revisions are summarized below.

**Reviewer’s Suggestion 1:**

 INTRODUCTION

 This study investigates the effect of the spin-off on NPF. The spin-off policy is a policy to improve the performance of Islamic banks in Indonesia. However, the author has not explained why NPF is used as a measure of the success of the spin-off policy. It needs to be explained why NPF is a serious problem in Islamic banking in Indonesia so that this spin-off policy can reduce financing risk.

**Authors’ response**: We have added information at the end of the introduction with the following narrative:

 “This is crucial given the pivotal role of financing risk management in impacting profitability, financing capacity, and overall financial stability (Pratami et al., 2023; Sriyana, 2015), where spin-offs hold the potential to mitigate such risks.”

**Reviewer’s Suggestion 2:**

 LITERATURE REVIEW

 Islamic bank financing is different from conventional bank financing. Islamic financing is broadly categorized as profit-loss sharing (PLS) financing and non-profit-sharing financing. PLS financings are subject to asymmetric information and moral hazard. As a result, PLS financing likely increases NPF. In this section, authors conduct the literature review regarding NPF but authors fail to address the impact of types of financing in Islamic banks on NPF. For example, see the following references :

 Warninda T., et al., (2019) [Ref-1]

 Sutrisno, S. et al., (2023) [Ref-2].

**Authors’ response**: Thank you for the positive feedback. We have added literature with the following narrative.

 “Furthermore, a high concentration of financing has been shown to negatively impact Non-Performing Financing (NPF) (Sutrisno et al., 2023). This underscores the necessity for a comprehensive understanding of the distinct risk profiles associated with various Islamic financing instruments in effective credit risk management. For instance, Warninda et al. (2019) found that, contrary to some assumptions, *Mudarabah* financing is not inherently riskier than *Musharakah* financing.”

**Reviewer’s Suggestion 3:**

 METHOD

 The difference and system GMM are a family of dynamic panel regression. The author must perform the robustness test with another estimation method. The author can employ static panel regression considering the number of banks is small (35 banks) while GMM requires a large number of banks.

**Authors’ response**: Thank you for your valuable feedback. We will certainly incorporate these suggestions into our future research. However, within the context of this current research, the progression from difference GMM estimation to system GMM is already considered part of the robustness check process. This is because the nature of a robustness check is to test whether the main results of an analysis remain consistent and not solely dependent on a single analytical approach, thus enhancing their reliability and generalizability. On the other hand, there are fundamental differences between the two estimations: difference GMM uses first differences to eliminate fixed effects, whereas system GMM combines instruments from both first differences and levels, allowing it to capture more of the variability in the data.

**Reviewer’s Suggestion 4:**

 RESULT AND DISCUSSION

 Authors need to perform some robustness tests:

 1. Changing variable of interest

 There is no single measure to calculate Islamic bank risk. To get robust results, the author needs to change the variable of interest. Financing loss provision (FLP) is an alternative measure of financing risk. For example, see the following reference:

 Widarjono A., et al., (2025) [Ref-3]

 2. Changing estimation method

 Considering that the number of small banks is 35, the author needs to estimate the model with static panel regression.

**Authors’ response**: Thank you for your valuable feedback. We will certainly incorporate these suggestions into our future research. However, within the context of this current research, the progression from difference GMM estimation to system GMM is already considered part of the robustness check process. This is because the nature of a robustness check is to test whether the main results of an analysis remain consistent and not solely dependent on a single analytical approach, thus enhancing their reliability and generalizability. On the other hand, there are fundamental differences between the two estimations: difference GMM uses first differences to eliminate fixed effects, whereas system GMM combines instruments from both first differences and levels, allowing it to capture more of the variability in the data.

**Reviewer’s Suggestion 5:**

 CONCLUSION

 In the conclusions, it is said that the spin-off policy is mandatory for every Islamic bank window. However, there is an important note from previous research that full spin-off likely decreases the performance of Islamic banks after several years of spin-off. See following references:

 Al Arif M., et al., (2020) [Ref-4]

 Trinugroho I, et al., 2021 [Ref-5]

**Authors’ responses**: Thank you for the feedback. It is true that several empirical studies reveal a decline in performance after spin-offs. However, from the perspective of financing risk management, we consider this policy to be viable. Moreover, with the issuance of the amendment to Law No. 21 of 2008 into Law No. 4 of 2023, which extends this policy, it increasingly indicates that this policy is not unfavorable. Nevertheless, we are refining the narrative in the conclusion as follows:

 “Consequently, the implementation of a spin-off policy represents a viable approach. This is further supported by the amendment of Law No. 21/2008 to Law No. 4/2023, which continues to accommodate spin-offs as a policy to foster the growth and contribution of Islamic banking to the national economy.”

# Reviewer report: Reviewer response for version 2

**DOI:** 10.5256/f1000research.176913.r372130

**Published:** 2025-04-12

**Authors:** Eko Suprayitno

**Affiliations:** 1 Universitas Islam Negeri Maulana Malik Ibrahim Malang, Malang, East Java, Indonesia

Abstract:

 1. Should provide a more concise summary of policy implications.

 2. Explain the role of GMM estimation more clearly.

 Introduction: 

 ��The justification for financing risk as the main focus should be stronger.

 ��The introduction is well-structured, presenting a logical flow from the importance of Islamic banking to the research gap.

 ��It could benefit from a stronger linkage between spin-offs and financing risk earlier in the section.

 �� Regulatory changes should be introduced earlier for better framing.

 Literature Review

- The literature review covers a broad range of studies, but it does not deeply analyze conflicting findings from past research.
- It could benefit from categorizing past studies (e.g., studies on spin-offs, studies on financing risk, and studies on policy implications).
- Needs a conceptual framework diagram to show research positioning.
- Could analyze contradictions in past research on non-performing financing (NPF).
- The section needs a conceptual framework diagram to clarify key variables and hypotheses.

Methods

 �� The methodology is rigorous and well-explained, but it assumes familiarity with GMM among readers.

 ��The rationale for choosing specific macroeconomic variables should be expanded.

 ��Justify why inflation and oil prices were included as control variables.

 ��Data collection methods are clearly documented, but more details on handling missing data would strengthen credibility. More details on how missing data was handled would strengthen credibility.

 Results and Discussion

 �� The interaction term (Spin-off × Size) needs a clearer interpretation.

 �� Managerial and policy implications should be expanded beyond statistical results.

 ��The empirical results are robust and align with previous studies, but some explanations are too technical for non-expert readers.

 ��The discussion connects well with the literature, but it could further explain why larger spin-off banks face higher risks.

 ��Clarification of Interaction Term (Spin-off × Size): The interpretation of why larger spin-off banks face higher risk needs more depth.

 ��Policy recommendations could be better integrated into this section rather than leaving them mostly in the conclusion.

 Conclusion

 �� Should differentiate theoretical contributions from practical implications.

 �� Future research suggestions are too general and need more specific directions.

 References

 �� Should add more recent studies (post-2022).

 �� Include cross-country comparisons to strengthen global relevance.

Is the work clearly and accurately presented and does it cite the current literature?

Yes

If applicable, is the statistical analysis and its interpretation appropriate?

Partly

Are all the source data underlying the results available to ensure full reproducibility?

Partly

Is the study design appropriate and is the work technically sound?

Partly

Are the conclusions drawn adequately supported by the results?

Partly

Are sufficient details of methods and analysis provided to allow replication by others?

Partly

Reviewer Expertise:

Islamic banking, Islamic macroeconomics, Islamic finance

I confirm that I have read this submission and believe that I have an appropriate level of expertise to confirm that it is of an acceptable scientific standard, however I have significant reservations, as outlined above.

# Response

**Published:** 2025-04-17

**Authors:** Zulfikar Bagus Pambuko

**Affiliations:** Islamic Economics Law, Universitas Muhammadiyah Magelang, Magelang, Central Java, Indonesia

Dear Reviewer

 We would like to warmly thank you for reading our manuscript and your valuable comments which would substantially improve our paper. We have followed carefully all your comments in the preparation of the revised version of the paper. Our revisions are summarized below.

**Reviewer’s Suggestion 1:**

 Abstract:

 1. Should provide a more concise summary of policy implications.

**Authors’ Response**: Improvements have been made to the conclusion sections.

  “… Therefore, spin-off policies for Islamic banks appear to be a viable strategy, as independence fosters enhanced risk sensitivity.”

 2. Explain the role of GMM estimation more clearly.

**Authors’ Response**: Improvements have been made to the methods sections.

  “… using a dynamic panel model with the Generalized Method of Moments (GMM) to overcome potential endogeneity issues that might bias the results.”

**Reviewer’s Suggestion 2:**

 Introduction:

1. The justification for financing risk as the main focus should be stronger.

**Authors’ Response**: We have added information to last paragraph

 “This is crucial given the pivotal role of financing risk management in impacting profitability, financing capacity, and overall financial stability (Pratami et al., 2023; Sriyana, 2015), where spin-offs hold the potential to mitigate such risks.”

1. The introduction is well-structured, presenting a logical flow from the importance of Islamic banking to the research gap.

**Authors’ Response**: Thank you

1. It could benefit from a stronger linkage between spin-offs and financing risk earlier in the section.

**Authors’ Response**: We have added information to last paragraph

 “This is crucial given the pivotal role of financing risk management in impacting profitability, financing capacity, and overall financial stability (Pratami et al., 2023; Sriyana, 2015), where spin-offs hold the potential to mitigate such risks.”

1. Regulatory changes should be introduced earlier for better framing.

**Authors’ Response**: We have added information to paragraph 2

 “The growing importance of this matter is underscored by the fact that the amendment of Law No. 21/2008 into Law No. 4/2023 retains spin-off as a key strategy for the acceleration of Islamic banking in Indonesia, albeit with the necessity of proper alignment.”

**Reviewer’s Suggestion 3:**

 Literature Review

1. The literature review covers a broad range of studies, but it does not deeply analyze conflicting findings from past research.
2. It could benefit from categorizing past studies (e.g., studies on spin-offs, studies on financing risk, and studies on policy implications).
3. Needs a conceptual framework diagram to show research positioning.
4. Could analyze contradictions in past research on non-performing financing (NPF).
5. The section needs a conceptual framework diagram to clarify key variables and hypotheses.

**Authors’ response**: We have added a new subsection (conceptual framework) to the literature review that addresses all the feedback as follows

*"Conceptual Framework*

 Although numerous factors have been identified as influencing financing risk, the findings across studies tend to show inconsistencies. These discrepancies may be attributed to differences in research settings, observation periods, and data analysis techniques. Nevertheless, this study focuses on presenting findings that are theoretically grounded and relevant to the existing literature.

 In line with this, the main objective of this research is to examine the impact of the spin-off decision on financing risk in Islamic banking in Indonesia—an aspect that has not been previously explored. The study also incorporates control variables, including internal bank conditions (bank size and market share) and macroeconomic indicators (inflation, economic growth, and oil price). The conceptual framework is illustrated in Figure 1, and the hypotheses are formulated as follows:

 H _1_: The spin-off decision has a negative effect on financing risk

 H _2_: Bank size has a positive effect on financing risk

 H _3_: Market share has a positive effect on financing risk

 H _4_: Inflation has a positive effect on financing risk

 H _5_: Economic growth has a negative effect on financing risk

 H _6_: Oil price has a negative effect on financing risk"

**Reviewer’s Suggestion 4:**

 Methods

1. The methodology is rigorous and well-explained, but it assumes familiarity with GMM among readers.
2. The rationale for choosing specific macroeconomic variables should be expanded.
3. Justify why inflation and oil prices were included as control variables.
4. Data collection methods are clearly documented, but more details on handling missing data would strengthen credibility. More details on how missing data was handled would strengthen credibility.

**Authors’ response: **Thank you for your feedback. We have provided a relatively detailed explanation regarding the use of GMM, covering its advantages, lag selection, instrument validity, etc. Furthermore, we offer the following additional clarification:

 “This approach was adopted to mitigate several underlying problems commonly encountered in panel data, specifically endogeneity, heteroskedasticity, and autocorrelation.”

 Furthermore, the selection of inflation and economic growth as macroeconomic variables was based on their common usage in the context of Islamic banking. Meanwhile, oil price was chosen because this variable is very rarely associated with Islamic banking performance, including financing risk.

 Subsequently, incomplete data were excluded from the analysis, leading us to employ an unbalanced panel data model. Consequently, only observations containing complete data were included in the estimation. This can be observed in Table 2 where the total data amounts to 1032 observations, while the final estimation in Table 4 only utilizes 948 observations.

**Reviewer’s Suggestion 5:**

 Results and Discussion

1. The interaction term (Spin-off × Size) needs a clearer interpretation.
2. Managerial and policy implications should be expanded beyond statistical results.
3. The empirical results are robust and align with previous studies, but some explanations are too technical for non-expert readers.
4. The discussion connects well with the literature, but it could further explain why larger spin-off banks face higher risks.
5. Clarification of Interaction Term (Spin-off × Size): The interpretation of why larger spin-off banks face higher risk needs more depth.
6. Policy recommendations could be better integrated into this section rather than leaving them mostly in the conclusion.

**Authors’ response**: We have refined the Discussion section to enhance its accessibility for a broader audience, including non-expert readers. Specifically, we have incorporated additional explanations to clarify the impact of the interaction variable (as detailed in the fifth paragraph) and macroeconomic factors (the seventh paragraph) on financing risk. This was done by providing more intuitive interpretations of the statistical findings, drawing analogies to real-world scenarios, and simplifying complex economic relationships without sacrificing the rigor of our analysis.

 “This is further supported by the significant positive effect of the interaction variable (spinoff*size), indicating that larger Islamic banks undergoing spin-offs face elevated financing risk compared to smaller ones. As highlighted by Trinugroho et al., (2021), the multifaceted nature of financing contracts in Islamic banking substantially elevates the risk of non-performing financing. Consequently, substantial asset expansion and spin-off implementation carry inherent risks that can be amplified by insufficient managerial readiness, making it harder to maintain financing quality.”

 “On the other hand, economic growth and inflation exhibit inconsistent results across different model specifications. Inflation generally exerts a positive influence on financing risk, as observed in models 2, 6, and 8. This finding aligns with previous studies, which suggests that inflation can reduce purchasing power, leading to increased loan defaults (Awdeh et al., 2024; Patiu & Eleazar, 2024). However, the impact of economic growth on financing risk is more nuanced, with negative effects observed in the difference GMM estimation and positive effects in the system GMM estimation. This discrepancy might be attributed to the non-linear relationship between economic growth and NPF. The difference GMM method may suggest short-term effects, potentially indicating that during periods of economic expansion, borrowers exhibit a higher propensity for timely repayment. Conversely, the system GMM method, which is considered to provide more robust estimates overall, may reveal longer-term effects. Over extended periods of strong economic growth, banks may exhibit increased lending appetite and potentially relax credit standards, leading to a higher incidence of future repayment difficulties (Castro, 2013; Huizinga & Laeven, 2019; Mongid et al., 2023). Furthermore, this divergence in results may be explained by the finite sample bias in difference GMM, which relies solely on the differenced data, discarding level information.”

 We acknowledge the reviewer's valuable suggestion to elaborate on policy recommendations within the discussion section to provide immediate context and justification. However, we have made a deliberate decision to retain the policy recommendations in the conclusion section. Our rationale for this placement is to ensure that the recommendations are presented as a direct and concise culmination of the entire analysis. By positioning them at the end, we aim to offer policymakers and other readers a readily accessible summary of the key actionable insights derived from our research. This structure allows readers to first grasp the full scope of our findings and their implications before focusing on the specific policy implications. We believe this approach enhances the clarity and impact of our recommendations as a direct takeaway from the study's overall conclusions.

**Reviewer’s Suggestion 6:**

 Conclusion

1. Should differentiate theoretical contributions from practical implications.

**Authors’ response**: We have refined the narrative, separating the empirical and theoretical contributions as follows:

 “Empirically, our findings reveal that Islamic windows carry a higher risk compared to full-fledged Islamic banks that undergo spin-offs. We also discover that spin-off Islamic banks with larger assets are more exposed to risk than those with smaller assets. This notice is supported by the regression that financing risk increases with the growth of assets and market share. Theoretically, this research contributes to the broader understanding of Islamic banking dynamics by providing empirical evidence on the risk implications of organizational structure transformation. Specifically, it highlights the potential risk reduction associated with the increased autonomy and focused operational framework of independent Islamic banks post-spin-off.”

1. Future research suggestions are too general and need more specific directions.

**Authors’ response**: We have refined the narrative as follows:

 “Furthermore, incorporating a broader range of internal (such as market concentration, market power, types of financing, operating expense ratio, etc) and external variables (exchange rate, money supply, etc), as well as expanding the analysis to include Islamic banks in countries that have implemented spin-offs, such as Saudi Arabia, Pakistan, Kuwait, etc., would provide a more comprehensive understanding of the phenomenon.”

**Reviewer’s Suggestion 7:**

 References

1. Should add more recent studies (post-2022).
2. Include cross-country comparisons to strengthen global relevance.

**Authors’ response**: Thank you for your constructive feedback. We have now incorporated additional relevant references, including cross-country studies and publications from 2022 onwards, to further strengthen the theoretical grounding and contextual relevance of our findings.

 Addou, K. I., Boulanouar, Z., Anwer, Z., Bensghir, A., & Ramadilli Mohammad, S. M. (2024). The impact of Basel III regulations on solvency and credit risk-taking behavior of Islamic banks. *International Journal of Islamic and Middle Eastern Finance and Management*, *17*(5), 915–935. https://doi.org/10.1108/IMEFM-05-2024-0248

 Nasir, M. S., Oktaviani, Y., & Andriyani, N. (2022). Determinants of Non-Performing Loans and Non-Performing Financing level: Evidence in Indonesia 2008-2021. *Banks and Bank Systems*, *17*(4), 116–128. https://doi.org/10.21511/bbs.17(4).2022.10

 Pratami, A., Afandi, A., Sriyana, J., & Feriyanto, N. (2023). The Role of Financing Models and Credit Risk on Islamic Bank Stability. *Cuadernos de Economía*, *46*(131), 43–53.

 Shah, S. A. A., Fianto, B. A., Sheikh, A. E., Sukmana, R., Kayani, U. N., & Bin Ridzuan, A. R. (2023). Role of fintech in credit risk management: an analysis of Islamic banks in Indonesia, Malaysia, UAE and Pakistan. *Journal of Science and Technology Policy Management*, *14*(6), 1128–1154. https://doi.org/10.1108/JSTPM-06-2022-0104

 Sriyana, J. (2015). Islamic banks’ profitability amid the competitive financing in Indonesia. *International Journal of Applied Business and Economic Research*, *13*(4), 1695–1710.

 Sutrisno, Widarjono, A., & Mohamad, M. (2023). Does Financing Diversification Improve Bank Risk? Evidence From Indonesian Islamic Rural Banks. *International Journal of Economics and Finance Studies*, *15*(4), 103–124. https://doi.org/10.34109/ijefs.202315406

 Warninda, T. D., Ekaputra, I. A., & Rokhim, R. (2019). Do Mudarabah and Musharakah financing impact Islamic Bank credit risk differently? *Research in International Business and Finance*, *49*, 166–175. https://doi.org/10.1016/j.ribaf.2019.03.002

# Reviewer report: Reviewer response for version 2

**DOI:** 10.5256/f1000research.176913.r364511

**Published:** 2025-02-06

**Authors:** Mutamimah Mutamimah

**Affiliations:** 1 Universitas Islam Sultan Agung, Semarang, Central Java, Indonesia

Ok, thanks for your revision. This suite with my suggestion.

 Good luck

Is the work clearly and accurately presented and does it cite the current literature?

Yes

If applicable, is the statistical analysis and its interpretation appropriate?

Yes

Are all the source data underlying the results available to ensure full reproducibility?

Yes

Is the study design appropriate and is the work technically sound?

Yes

Are the conclusions drawn adequately supported by the results?

Yes

Are sufficient details of methods and analysis provided to allow replication by others?

Yes

Reviewer Expertise:

Economics, Management, Financial Management

I confirm that I have read this submission and believe that I have an appropriate level of expertise to confirm that it is of an acceptable scientific standard.

# Reviewer report: Reviewer response for version 1

**DOI:** 10.5256/f1000research.172879.r353263

**Published:** 2025-01-10

**Authors:** Mutamimah Mutamimah

**Affiliations:** 1 Universitas Islam Sultan Agung, Semarang, Central Java, Indonesia

The article is well written and meets the rules for good articles. The problem formulation is based on research gaps from previous research results. Apart from that, the research method is also appropriate, including the indicators, and statistical analysis and results.

 However, researchers need to attach the year in which the Islamic banking spin-off was carried out, because each Islamic banking spin-off is different.

 In table 2 about Descriptive analysis of variables, the description of each variable should be explained well.

 Furthermore, in conclusion,  it must have limitations and future research agenda. It would be good to explain why the research did not analyze financing risks before the spin-off, so there will be a difference between before the spin-off and after the spin-off.

 Good luck!

Is the work clearly and accurately presented and does it cite the current literature?

Yes

If applicable, is the statistical analysis and its interpretation appropriate?

Yes

Are all the source data underlying the results available to ensure full reproducibility?

Yes

Is the study design appropriate and is the work technically sound?

Yes

Are the conclusions drawn adequately supported by the results?

Yes

Are sufficient details of methods and analysis provided to allow replication by others?

Yes

Reviewer Expertise:

Economics, Management, Financial Management

I confirm that I have read this submission and believe that I have an appropriate level of expertise to confirm that it is of an acceptable scientific standard, however I have significant reservations, as outlined above.

# Response

**Published:** 2025-01-20

**Authors:** Zulfikar Bagus Pambuko

**Affiliations:** Islamic Economics Law, Universitas Muhammadiyah Magelang, Magelang, Central Java, Indonesia

We would like to warmly thank you for reading our manuscript and your valuable comments which would substantially improve our paper. We have followed carefully all your comments in the preparation of the revised version of the paper. Our revisions are summarized below.

**Reviewer’s Suggestion 1:** The article is well written and meets the rules for good articles. The problem formulation is based on research gaps from previous research results. Apart from that, the research method is also appropriate, including the indicators, and statistical analysis and results. However, researchers need to attach the year in which the Islamic banking spin-off was carried out, because each Islamic banking spin-off is different.           

**Authors’ Response:** Following your insightful suggestion, we have revised the Method section to provide a more comprehensive overview of the restructuring processes within the Indonesian Islamic banking industry. The newly added paragraph, which details historical data on spin-offs, conversions, and mergers, directly addresses your feedback regarding the need for more specific examples.

 “The Indonesian Islamic banking landscape has witnessed various restructuring strategies. Pure spin-offs were employed by banks such as Bukopin Syariah (2008), BNI Syariah (2008), BRI Syariah (2010), BJB Syariah (2010), and Bank Nano Syariah (2024). Conversion, another common approach, was adopted by BTPN Syariah (2014), Bank Aceh Syariah (2016), BPD NTB Syariah (2018), and BPD Riau Kepri Syariah (2022). The formation of Bank Syariah Indonesia in 2021 marked a significant merger in the sector. Moreover, a combination of acquisition and conversion was utilized sequentially by banks including Bank Panin Dubai Syariah (2009), Bank Victoria Syariah (2010), BCA Syariah (2010), and Bank Aladin Syariah (2010).”

**Reviewer’s Suggestion 2:** In table 2 about Descriptive analysis of variables, the description of each variable should be explained well.           

**Authors’ Response:** Thank you for your valuable feedback. We have addressed your concerns regarding variable descriptions in the descriptive analysis.

 “Table 2 presents descriptive statistics for the variables spanning from 2006 to 2022. The average value of NPF, as a financing risk proxy, stands at 3.10%, remaining below the generally accepted upper limit of 5.00%. The lowest NPF value, at 0.00%, indicates that some Islamic banks exhibit no risky financing, while the highest recorded NPF value is 53.27%. This relatively high variability is supported by a standard deviation of 4.17%. SPINOFF, a dummy variable, has a mean of 0.27, indicating that spin-off events are relatively rare. Its standard deviation of 0.45 suggests moderate variability in occurrence. Regarding assets and market share, the lower mean values compared to the standard deviation suggest that Islamic banks in Indonesia are still dominated by a few larger banks, reflecting differences in institutional capacity and market reach.

 Similarly, the Consumer Price Index (CPI) and Industrial Production Index (IPI) show moderate variability, with standard deviations of 2.70 and 18.33, respectively, reflecting macroeconomic conditions that influence Islamic banking performance. Oil prices (OIL), a critical variable for resource-dependent economies, have a mean of IDR 0.86 million per barrel, with relatively stable fluctuations (standard deviation of 0.28). This stability contrasts with the high variability observed in institutional size and non-performing financing.

 Overall, the descriptive analysis underscores significant heterogeneity in institutional characteristics and economic factors, suggesting the need for tailored strategies in managing Islamic banks. Institutions must address wide disparities in size and market share, ensuring resilience to external economic conditions such as inflation, industrial output fluctuations, and oil price volatility.”

**Reviewer’s Suggestion 3:** Furthermore, in conclusion, it must have limitations and future research agenda. It would be good to explain why the research did not analyze financing risks before the spin-off, so there will be a difference between before the spin-off and after the spin-off. Good luck!

**Authors’ Response:** We appreciate your insightful comments. We have added the limitations and future research opportunities to the conclusion section.

 “The present study has identified several limitations. The utilization of a dummy variable to assess the impact of spin-offs on financing risk, while effective in capturing the immediate effects of regulatory policies, may not fully account for the nuanced dynamics at play. Moreover, the study's scope is constrained by the use of semi-annual data up to 2022 and its focus on the Indonesian Islamic banking industry. To address these limitations, future research could benefit from employing a difference-in-differences analysis to more precisely estimate the causal impact of spin-offs. Furthermore, incorporating a broader range of internal and external variables, as well as expanding the analysis to include Islamic banks in Southeast Asia or OIC countries, would provide a more comprehensive understanding of the phenomenon.”

# Reviewer report: Reviewer response for version 1

**DOI:** 10.5256/f1000research.172879.r336316

**Published:** 2024-11-07

**Authors:** Azharsyah Ibrahim

**Affiliations:** 1 Universitas Islam Negeri Ar-Raniry, Banda Aceh, Indonesia

**Summary**

 This paper investigates the impact of the spin-off decision (Islamic windows separating from conventional banks to become independent Islamic banks) on financing risk in Indonesian Islamic banks. Using semi-annual data from 2006-2022 for 35 Islamic banks, the study employs a dynamic panel model with Generalized Method of Moments (GMM) estimation. The key finding is that spin-offs significantly reduce financing risk, particularly for larger banks. The study also finds that larger Islamic banks and those with a higher market share generally face higher financing risk. The negative correlation between oil prices and non-performing financing suggests that increasing oil prices may indirectly benefit Islamic banks by stimulating the economy.

**Evaluation**

- **Is the work clearly and accurately presented and does it cite the current literature?** Partly. While the presentation is generally clear, some areas could benefit from more precise language and further elaboration. The literature review, while extensive, could be structured more thematically to better highlight the existing research gaps and the study's contribution. Some cited works appear very recent (2023-2024) and I am unable to verify their accessibility/existence given my current knowledge cutoff. It's important to ensure all cited works are publicly accessible.
- **Is the study design appropriate and is the work technically sound?** Partly. The use of a dynamic panel model with GMM is appropriate for addressing potential endogeneity. However, the justification for using the OPEC basket price as a sole indicator of global oil prices could be strengthened. A broader measure or a combination of indicators might be more representative. Additionally, more detail on the specific GMM implementation (e.g., lag selection, instrument validity tests beyond Sargan) would enhance the technical soundness.
- **Are sufficient details of methods and analysis provided to allow replication by others?** Partly. While the data sources are mentioned, the specific variables used within the model (e.g., how "market share" is calculated) could be more explicitly defined. The provided dataset link on Zenodo was checked and is accessible. However, clear instructions on how to merge the bank-specific data with the macroeconomic data are necessary for full replication. More detail on the GMM implementation is also needed (as mentioned above).
- **If applicable, is the statistical analysis and its interpretation appropriate?** Partly. The interpretation of the results generally aligns with the findings. However, the discussion of the inconsistent results for economic growth and inflation could be improved. Exploring potential reasons for these inconsistencies or considering alternative model specifications might provide more robust insights. Furthermore, while the Sargan test is reported, more comprehensive diagnostics related to the GMM estimation would strengthen the analysis.
- **Are all the source data underlying the results available to ensure full reproducibility?** Yes. The data sources are listed and the Zenodo link provides the bank-level data. The macroeconomic data sources are also publicly accessible. However, as noted above, clearer instructions on data merging are needed.
- **Are the conclusions drawn adequately supported by the results?** Partly. The main conclusion regarding the positive impact of spin-offs on reducing financing risk is supported by the findings. However, the claim that larger spin-off banks are *more* exposed to risk needs further clarification. While the results show that size increases risk in general, it's not entirely clear from the presented analysis whether this effect is amplified *after* the spin-off, or if larger banks simply start at a higher risk level pre-spin-off. More nuanced analysis and discussion are needed to fully support this specific conclusion.

**Specific Recommendations for Improvement:**

- **Literature Review:** Organize the literature thematically and clearly articulate the research gap being addressed. Verify the accessibility of all cited works.
- **Methodology:** Provide a stronger justification for the choice of oil price indicator. Elaborate on the specific GMM implementation details (lag selection, instrument details, and robustness checks).
- **Data and Replication:** Provide clear definitions of all variables, including calculations. Include detailed instructions on how to merge the datasets used.
- **Statistical Analysis:** Explore reasons for the inconsistent results of control variables. Provide more comprehensive GMM diagnostics.
- **Conclusion:** Refine the discussion about the impact of size on risk for spin-off banks. Provide clearer evidence to support the claim of increased risk for larger spin-off banks compared to smaller ones. Consider separating the impact of size on risk in general from the specific impact on spin-off banks.

Is the work clearly and accurately presented and does it cite the current literature?

Partly

If applicable, is the statistical analysis and its interpretation appropriate?

Partly

Are all the source data underlying the results available to ensure full reproducibility?

Yes

Is the study design appropriate and is the work technically sound?

Partly

Are the conclusions drawn adequately supported by the results?

Partly

Are sufficient details of methods and analysis provided to allow replication by others?

Partly

Reviewer Expertise:

Islamic management, banking, and finance

I confirm that I have read this submission and believe that I have an appropriate level of expertise to confirm that it is of an acceptable scientific standard, however I have significant reservations, as outlined above.

# Response

**Published:** 2025-01-20

**Authors:** Zulfikar Bagus Pambuko

**Affiliations:** Islamic Economics Law, Universitas Muhammadiyah Magelang, Magelang, Central Java, Indonesia

We would like to warmly thank you for reading our manuscript and your valuable comments which would substantially improve our paper. We have followed carefully all your comments in the preparation of the revised version of the paper. Our revisions are summarized below.

**Reviewer’s Suggestion 1:** Is the work clearly and accurately presented and does it cite the current literature? Partly. While the presentation is generally clear, some areas could benefit from more precise language and further elaboration. The literature review, while extensive, could be structured more thematically to better highlight the existing research gaps and the study's contribution. Some cited works appear very recent (2023-2024) and I am unable to verify their accessibility/existence given my current knowledge cutoff. It's important to ensure all cited works are publicly accessible.

**Authors’ Response: **We would like to express our gratitude for your valuable feedback in enhancing the quality of our article. In response to your suggestion, we have incorporated a dedicated section on literature review as follows:

 “ **Literature Review**

 The management of bad financing or non-performing financing (NPF) presents a critical challenge for Islamic banking institutions in Indonesia (Imaduddin, 2008; Nugroho et al., 2018; Soekapdjo et al., 2018). Non-performing financing, characterized by loans or financing contracts in default or at risk of default, can have far-reaching implications on the stability and credibility of financial institutions (Hernawati et al., 2021). In traditional banking, its known as non-performing loan (NPL). The existence of financing risk can hinder the stability of Islamic banking entities, potentially jeopardizing their capacity to facilitate economic growth and foster financial inclusivity (Banna et al., 2022). Considering the distinct characteristics of Islamic finance, rooted in risk-sharing and asset-backed principles, a comprehensive exploration of how spin-off decisions influence financing risk, specifically non-performing financing, requires further investigation.

*Bank-specific factors influencing financing risk*

 Research on NPF in Islamic banking and NPL in conventional banking reveals that various internal and external factors influence financing risk. For conventional banks, external factors such as macroeconomic conditions play a dominant role in shaping NPL levels, while in Islamic banks, both internal and external factors significantly affect NPF (Loang et al., 2023; Setiawan et al., 2017). Among bank-specific factors, an increase in NPF is associated with variables such as bank size, operating efficiency ratio, liquid asset ratio, financing-to-deposit ratio, and foreign ownership (Havidz & Setiawan, 2015; Hosen & Muhari, 2019; Osunkoya et al., 2023; Rahim & Zakaria, 2013; Saw et al., 2022; Zheng et al., 2019). Conversely, factors that contribute to a reduction in NPF include higher net operating profit, capital adequacy ratio, return on assets, cost-to-income ratio, deposit fund growth, return on equity, loan growth, and market concentration (Hartanto & Samputra, 2023; Hosen & Muhari, 2019; Muhammad et al., 2020; Rahim & Zakaria, 2013; Zheng et al., 2019).

*Macroeconomic factors influencing financing risk*

 Macroeconomic conditions also significantly impact the level of financing risk in Islamic banks. Negative economic indicators such as exchange rate fluctuations, inflation, and economic policy uncertainty have been found to increase NPF levels (Firmansyah, 2014; Hosen & Muhari, 2019; Karadima & Louri, 2021; Osunkoya et al., 2023; Zheng et al., 2019). On the other hand, positive macroeconomic trends such as GDP growth and reduced unemployment contribute to a decrease in NPF (Ferhi, 2018; Hassan et al., 2019; Hernawati et al., 2021; Le & Diep, 2020; Zheng et al., 2019). Additionally, a surplus in foreign exchange reserves can trigger a financing boom, which may subsequently affect financing quality and contribute to increased NPF levels (Kuncoro, 2024).”

**Reviewer’s Suggestion 2:** Is the study design appropriate and is the work technically sound? Partly. The use of a dynamic panel model with GMM is appropriate for addressing potential endogeneity. However, the justification for using the OPEC basket price as a sole indicator of global oil prices could be strengthened. A broader measure or a combination of indicators might be more representative. Additionally, more detail on the specific GMM implementation (e.g., lag selection, instrument validity tests beyond Sargan) would enhance the technical soundness.

**Authors’ Response:**

 We have added an explanation regarding the use of the OPEC basket price in the “Method” section.

 “This price serves as a crucial benchmark in determining crude oil prices in the global market, including Indonesia, although it is merely one of many indicators influenced by various complex factors.”

 Additionally, we have provided a more detailed explanation of lag selection in GMM estimation.

 “A lag order of one (lag 1) was used for estimation”

**Reviewer’s Suggestion 3:** Are sufficient details of methods and analysis provided to allow replication by others? Partly. While the data sources are mentioned, the specific variables used within the model (e.g., how "market share" is calculated) could be more explicitly defined. The provided dataset link on Zenodo was checked and is accessible. However, clear instructions on how to merge the bank-specific data with the macroeconomic data are necessary for full replication. More detail on the GMM implementation is also needed (as mentioned above).

**Authors’ Response: **Thank you for the suggestion. Supplementary information regarding the variables has been provided in Table 1.

 “Non-performing financing is the ratio of bad financing by total financing”

 “Inflation is measured using the Consumer Price Index (CPI), which compares the total cost of a fixed basket of goods and services in the current period to the cost of the same basket in a base period”

 “Economic growth is measured using the Industrial Production Index (IPI), which is calculated by dividing the value of production in a specific period by the value of production in the base year 2010.”

 The data used in this study is a combination of panel data (internal data from Islamic banks) and time series data (macroeconomic data). For the macroeconomic data, we ensured that the estimation periods aligned, and duplicated the time series data for each Islamic bank observation. Given the unbalanced nature of the panel data, macroeconomic data was only added to complete Islamic bank observations.

**Reviewer’s Suggestion 4:** If applicable, is the statistical analysis and its interpretation appropriate? Partly. The interpretation of the results generally aligns with the findings. However, the discussion of the inconsistent results for economic growth and inflation could be improved. Exploring potential reasons for these inconsistencies or considering alternative model specifications might provide more robust insights. Furthermore, while the Sargan test is reported, more comprehensive diagnostics related to the GMM estimation would strengthen the analysis.

**Authors’ Response: **Following the reviewer’s suggestion, we have enhanced the discussion of economic growth and inflation in the “empirical results” section. Furthermore, we have provided a more detailed statistical analysis to elucidate the differences between the difference GMM and system GMM estimators.

 “On the other hand, economic growth and inflation exhibit inconsistent results across different model specifications. Inflation generally exerts a positive influence on financing risk, as observed in models 2, 6, and 8. This finding aligns with previous studies, which suggests that inflation can reduce purchasing power, leading to increased loan defaults (Awdeh et al., 2024; Patiu & Eleazar, 2024). However, the impact of economic growth on financing risk is more nuanced, with negative effects observed in the difference GMM estimation and positive effects in the system GMM estimation. This discrepancy might be attributed to the non-linear relationship between economic growth and NPF, where difference GMM captures short-term effects while system GMM captures long-term effects. The positive relationship in the system GMM, which is considered to provide more robust estimates, may reflect the potential for credit booms during periods of economic expansion, leading Islamic banks to loosen lending standards (Castro, 2013; Huizinga & Laeven, 2019; Mongid et al., 2023). Furthermore, this divergence in results may be explained by the finite sample bias in difference GMM, which relies solely on differences across time periods and excludes the level data.”

**Reviewer’s Suggestion 5:** Are all the source data underlying the results available to ensure full reproducibility? Yes. The data sources are listed and the Zenodo link provides the bank-level data. The macroeconomic data sources are also publicly accessible. However, as noted above, clearer instructions on data merging are needed.

**Authors’ Response: **Thank you for the suggestion. The data used in this study is a combination of panel data (internal data from Islamic banks) and time series data (macroeconomic data). For the macroeconomic data, we ensured that the estimation periods aligned, and duplicated the time series data for each Islamic bank observation. Given the unbalanced nature of the panel data, macroeconomic data was only added to complete Islamic bank observations.

**Reviewer’s Suggestion 6:** Are the conclusions drawn adequately supported by the results? Partly. The main conclusion regarding the positive impact of spin-offs on reducing financing risk is supported by the findings. However, the claim that larger spin-off banks are more exposed to risk needs further clarification. While the results show that size increases risk in general, it's not entirely clear from the presented analysis whether this effect is amplified after the spin-off, or if larger banks simply start at a higher risk level pre-spin-off. More nuanced analysis and discussion are needed to fully support this specific conclusion.

**Authors’ Response: **The conclusion that larger spin-off banks are more exposed to risk is derived from the interaction variables between the spin-off and size variables (spinoff*size), as detailed in the fifth paragraph of the "Empirical Results" section.

 “Furthermore, these results are corroborated by the estimations of the interaction variable (spinoff*size), which significantly influences non-performing financing. Thus, larger Islamic banks that undergo spin-offs carry a higher financing risk compared to smaller banks. As highlighted by Trinugroho et al., (2021), the multifaceted nature of financing contracts in Islamic banking substantially elevates the risk of non-performing financing.”

 Specific Recommendations for Improvement:

**Reviewer’s Suggestion 7:** Literature Review: Organize the literature thematically and clearly articulate the research gap being addressed. Verify the accessibility of all cited works.

**Authors’ Response: **Please refer to response number 1.

**Reviewer’s Suggestion 8:** Methodology: Provide a stronger justification for the choice of oil price indicator. Elaborate on the specific GMM implementation details (lag selection, instrument details, and robustness checks).

**Authors’ Response: **Please refer to response number 2.

**Reviewer’s Suggestion 9:** Data and Replication: Provide clear definitions of all variables, including calculations. Include detailed instructions on how to merge the datasets used.

**Authors’ Response: **Please refer to response number 4.

**Reviewer’s Suggestion 10:** Statistical Analysis: Explore reasons for the inconsistent results of control variables. Provide more comprehensive GMM diagnostics.

**Authors’ Response: **Based on the literature review, our implementation of the GMM model adheres to the established guidelines. While inconsistencies in the estimation results between difference GMM and system GMM for some variables are common, the inconsistencies in the spin-off, size, and IPI variables might be attributed to endogeneity issues better addressed by system GMM or measurement errors in difference GMM. This can be explained by the inherent differences between the two estimators. Difference GMM uses first differences to eliminate fixed effects, whereas system GMM combines instruments from both first differences and levels, allowing it to capture more of the variability in the data (The preceding explanation offers a more in-depth analysis of response number 4).

**Reviewer’s Suggestion 11:** Conclusion: Refine the discussion about the impact of size on risk for spin-off banks. Provide clearer evidence to support the claim of increased risk for larger spin-off banks compared to smaller ones. Consider separating the impact of size on risk in general from the specific impact on spin-off banks.

**Authors’ Response: **we have explained separately in the last 2 paragraphs in the Results and Discussion section related to the general impact of size on NPF, followed by a specific examination of the interaction between spin-off and size on NPF. The positive interaction term between spin-off and size (spinoff*size) on Table 4 suggests an increased risk of NPF for larger spin-off banks. we have also added a supporting statement, “As highlighted by Trinugroho et al., (2021), the multifaceted nature of financing contracts in Islamic banking substantially elevates the risk of non-performing financing.”
